# Effect of Time after Anterior Cruciate Ligament Tears on Proprioception and Postural Stability

**DOI:** 10.1371/journal.pone.0139038

**Published:** 2015-09-30

**Authors:** Dae-Hee Lee, Jin-Hyuck Lee, Sung-Eun Ahn, Min-Ji Park

**Affiliations:** 1 Department of Orthopaedic Surgery, Samsung Medical Center, Sungkyunkwan University School of Medicine, Seoul, Korea; 2 Department of Sports Medical Center, Korea University College of Medicine, Anam Hospital, Seoul, Korea; Victoria University, AUSTRALIA

## Abstract

This study was designed to compare proprioception and postural stability in patients with acute (time from injury ≤ 3 months) and chronic (time from injury > 3 months) ACL tears, and to evaluate the correlation between time interval after ACL injury and proprioception. Thigh muscle strength, postural stability, and joint position sense were compared in 48 patients with acute ACL tears and in 28 with chronic ACL tears. Maximal torque (60°/sec) of the quadriceps and hamstring was evaluated using an isokinetic testing device. Postural stability was determined from the anterior-posterior (APSI), medial-lateral (MLSI), and overall (OSI) stability indices using stabilometry. Joint position sense was also tested by reproduction of passive positioning (RPP). Muscle strengths and stability indices on both the involved and uninvolved sides were similar in the acute and chronic ACL tear groups. RPP on the involved side was significantly greater in the chronic than in the acute ACL tear group (7.8° vs. 5.6°, P = 0.041). Two of three stability indices (APSI, OSI) and RPP were significantly greater on the involved than the uninvolved side in the chronic ACL tear group.

## Introduction

The femoral and tibial attachment sites of the anterior cruciate ligament (ACL) contain mechanoreceptors such as the Pacinian corpuscles, Ruffini endings, and Golgi tendon organ-like corpuscles,[1, 2] all of which play a role in proprioception. Therefore, ACL tears not only create mechanical instability, they may also impair proprioception, including postural stability, because disruption of the ACL may lead to a lack of afferent sensory input from mechanoreceptors to the central nervous system.[3–5] Several studies have shown a significant loss in proprioception in ACL-deficient knees.[6–8] Single limb stance postural stability has also been reported to be impaired in patients with isolated ACL tears.[9, 10]

Proprioception, including postural stability, may be influenced by a variety of factors, including age, muscle weakness, level of physical activity, and previous injury to the lower-extremities.[11–13] In addition, elapsed time from the injury may affect proprioception and postural stability.[14–16] For example, a histologic study investigating the fate of mechanoreceptors present in ruptured ACLs reported an inverse relationship between the elapsed time from injury and the number of mechanoreceptors in the torn ACL remnant. To date, however, few clinical studies have evaluated the relationships between time after ACL tear and proprioception and postural stability or have compared proprioception and postural stability in patients with acute and chronic ACL tears.

This study was therefore designed to compare the proprioception and postural stability of patients with acute (time from injury ≤ 3 months) and chronic (time from injury > 3 months) ACL tears, and to evaluate the correlation between time interval after ACL injury and proprioception and postural stability. It was hypothesized that patients with chronic ACL tears would have decreased proprioception and postural stability.

## Materials and Methods

### Ethics Statement

The ethical approval of this study protocol was granted by Institutional Review Board of the Korea University Anam Hospital (permit no. AN10010-001). Written informed consent was obtained from all subjects before participation in this study (parental/guardian consent was obtained for minors).

### Patient Selection and Study Design

This prospective longitudinal trial enrolled all candidates for ACL reconstruction with isolated primary ACL ruptures confirmed by magnetic resonance imaging (MRI) and physical examinations, such as positive anterior drawer, Lachmann, and/or pivot shift tests (more than grade II). Patients with concomitant meniscus tear were excluded to eliminate bias resulting from meniscus tear. Also excluded were patients with bilateral ACL injuries or associated injuries to any other ligament (i.e., the medial or lateral collateral ligament or the posterior cruciate ligament), previous injury / surgery to either knee, or any associated extra-articular lesions. Patients were also excluded if they were unable to perform the isokinetic muscle strength, postural stability, or proprioception tests due to pain or limited motion of the knee joint due to effusion. Patients who underwent surgery less than 3 months after injury were categorized as having acute ACL tears, whereas those who underwent surgery after 3 months were categorized as having chronic ACL tears. Of the 82 patients (82 knees) approached, 80 agreed to take part in the study. After assessments for eligibility, 76 patients, 48 with acute and 28 with chronic ACL tears, were enrolled. The baseline demographic characteristics of the two groups were similar except for time interval from injury to surgery (Table 1).

**Table 1:** Demographic characteristics of subjects with acute and chronic anterior cruciate ligament tears.

	Acute ACL group	Chronic ACL group	*P*-value
Sample size (number)	48	28	
Gender (male/female)	42/6	24/4	
Age (years)	32.1 ± 10.8 (16 to 55)	34.7 ± 12.7 (17 to 58)	0.316
Height (cm)	171.3 ± 7.4 (149 to 187)	171.0 ± 6.5 (157 to 183)	0.855
Weight (kg)	70.3 ± 11.6 (43 to 110)	71.9 ± 10.7 (51 to 97)	0.562
Body mass index (kg/m^2^)	23.8 ± 3.2 (17.7 to 31.7)	24.6 ± 3.3 (19.2 to 32.1)	0.396
Time interval from trauma (months)	1.1 ± 0.19 (1 to 2)	6.8 ± 4.3 (3 to 24)	**<0.001** *

ACL, anterior cruciate ligament

Results reported as mean ± SD (range)

*P< 0.05

### Tests of Isokinetic Strength, Postural Stability, and Proprioception

Isokinetic knee extension/flexion (concentric/concentric muscle contraction) strength was measured with each subject seated on a Biodex multi-joint system 4 (Biodex Medical Systems, Shirley, NY) with his/her trunk perpendicular to the floor, and hips and knees flexed to 90 degrees. The center of motion of the lever arm was aligned as accurately as possible with the lateral femoral condyle of the knee being tested. A strap was used to immobilize each subject’s thigh, and the dynamometer attachment was aligned to the lateral malleolus of the lower leg of the knee being tested. The resistance pad was placed as distally as possible on the tibia while still allowing full dorsiflexion at the ankle. Before each test session, each individual performed a set of 5 warm-up submaximal knee flexions and extensions of each leg at 60 degrees/sec. Each test session consisted of 5 isokinetic knee extensions and flexions (range of motion, 80 to 0 degrees) of each leg at 60 degrees/sec, with a rest time of 30 seconds between tests. Peak flexion and extension torques were recorded (Nm/kg). Extensor strength was regarded as quadriceps strength, and flexor strength was regarded as hamstring muscle strength. The mean value of two trials was regarded as the maximal peak torque of the hamstring and quadriceps.

Postural stability tests were performed using the Biodex Stability System (BSS; Biodex Medical Systems), with a movable balance platform that provided up to 20° of surface tilt in a 360° range of motion. This platform, which interfaced with computer software (Biodex, Version 1.32), enabled the device to objectively assess balance. Participants were instructed to stand with a bare foot on the BSS locked platform, to keep the other foot off the ground in a comfortable position, to keep their arms at their sides and to look straight ahead at a point on the wall approximately 1 m away at eye level. As soon as the subject was able to maintain the point indicating that his/her location was on the center of pressure, the examiner recorded the foot location using a coordinate system consisting of the lateral malleolus and the heel cord on the foot plate. After positioning, subjects were instructed to maintain the same position of their feet until the end of each test. Subjects unable to maintain balance during testing were allowed to briefly touch their toes with the opposite foot or grasp the handrails for a short time to re-establish balance as soon as possible. If a subject was unable to quickly re-establish balance, that test was cancelled. Each test consisted of two trials, starting at level 12 (most stable) and gradually decreasing to level one (least stable), with the stability level automatically declining every 1.66 sec. Two test evaluations of 20 sec each were performed, with 10 sec between each pair of tests. The mean and standard deviation of the two trials was calculated by the stability system. The measures of balance and postural stability included anterior-posterior, medial-lateral, and overall stability index scores. A lower stability index was associated with a more stable platform, indicating greater dynamic balance or postural stability of the subject.

A reproduction of passive positioning (RPP) using the Biodex multi-joint system 4 was used to measure the joint position sense of knee joint proprioception.The mean and standard deviation of the two trials was calculated by this system. This system could measure the RPP to two decimal places.

The subject was seated on the isokinetic dynamometer chair with eyes closed, with the hips and knees flexed to 90°. The knee joint was then moved into a predetermined amount of extension (45° of knee flexion in our study) and held for 5 seconds, with each subject instructed to remember that target position. The knee was then passively returned to the starting position (90° of knee flexion). The knee was moved by the Biodex system, and the subject was asked to push a switch when he/she thought that the angle of knee joint reached the previous target angle (45° of knee flexion). The difference between the angle indicated by the patient and the target angle was recorded. Tests were performed twice on both legs alternately, with 30 seconds of resting time between tests. For both the postural stability tests and the RPP tests, the uninjured knee was tested first.

### Statistical analysis

An RPP difference > 1° between the acute and chronic ACL tear groups was regarded as clinically significant. An a priori power analysis was performed to determine the sample size using a two-sided hypothesis test at an alpha level of 0.05 and a power of 0.8. The results of a pilot study involving 5 knees in each group indicated that 25 knees would be required to detect a significant between-group RPP difference of > 1°, the primary outcome measure. Forty eight patients with acute and 28 with chronic ACL tears were assessed. Overall, the power of this study was 0.851 for detecting a significant between group difference in RPP.

To quantify the test-retest reliability of isokinetic strength, postural stability and joint position sense, intraclass correlation coefficients (ICCs) were calculated for two trials of maximal peak torques of the quadriceps and hamstring. ICCs were also calculated for two measurements of each stability index, including anterior-posterior, medial-lateral, and overall stability indices, and RPP. ICC values >0.75, between 0.4 and 0.75, and <0.4 represented good, fair, and poor reliability/accuracy, respectively. [17]

Mean values of the stability indices, strengths of the hamstring and quadriceps muscles, their ratio, and joint position sense (RPP) were compared in the acute and chronic ACL tear groups, and on the uninvolved and involved sides, using Student’s t-tests or Mann-Whitney U tests, as appropriate. Correlations between joint position sense and stability index, muscle strength, and demographic characteristics, including time interval from injury, were assessed by Pearson correlation analysis. Multiple linear regression analysis was performed to identify variables that independently affected joint position sense on the involved side. Five parameters, specifically age, duration from injury to surgery, uninvolved and involved hamstring torques, and RPP of the uninvolved knee, were included as independent variables and RPP of the involved side was defined as the dependent variable. Dataset used in this study is given in S1 Dataset. All statistical analyses were performed using IBM SPSS Statistics version 20 software (IBM Corporation, USA), with a *P* value <0.05 considered statistically significant.

## Results

In all subjects, the test-retest reliabilities of isokinetic peak torque were acceptable for the quadriceps (ICC = 0.82) and hamstring (ICC = 0.79) muscles. In addition, the test-retest reliabilities for postural stability were good for overall (ICC = 0.76), anterior-posterior (ICC = 0.77), and medial-lateral (ICC = 0.75) stability indices, as well as for RPP (ICC = 0.78).

### Muscle Strength and Hamstring to Quadriceps (HQ) Ratio

There were no significant differences between the acute and chronic ACL tear groups in isokinetic maximal peak torques of the quadriceps and hamstring muscles at 60°/sec, on both the involved and uninvolved sides. Moreover, there were no between group differences in hamstring to quadriceps ratio on either side (Table 2).

**Table 2:** Muscle strength and hamstring/quadriceps ratios of subjects with acute and chronic anterior cruciate ligament tears.

	Acute ACL group	Chronic ACL group	*P*-value
Quadriceps torque (uninvolved)	195.5 ± 51.2	203.7 ± 42.7	0.511
Quadriceps torque (involved)	116.1 ± 53.1	124.9 ± 62.1	0.535
Hamstring torque (uninvolved)	89.6 ± 25.8	90.7 ±35.0	0.874
Hamstring torque (involved)	53.8 ± 26.1	58.0 ± 29.6	0.547
HQ ratio (uninvolved)	46.7 ± 11.3	46.6 ± 16.0	0.982
HQ ratio (involved)	47.1 ± 16.9	47.7 ± 11.7	0.894

ACL, anterior cruciate ligament; H, hamstring; Q, quadriceps

Results reported as mean ± SD. Muscle strength was calculated as isokinetic peak torque of the quadriceps and hamstring muscles at 60°/sec (Nm/kg).

### Stability Indices and Reproduction of Passive Positioning

**Fig 1:**
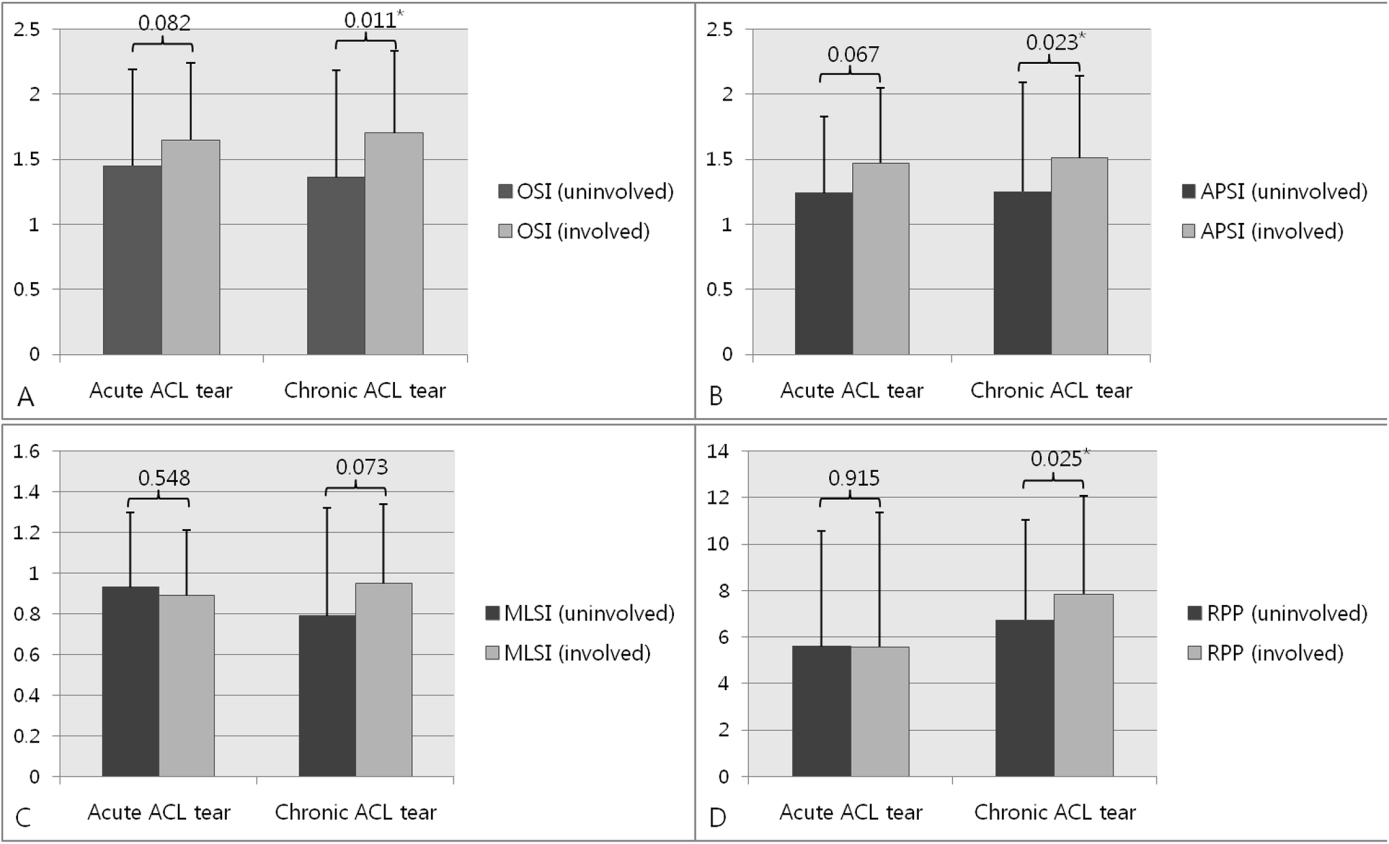
Postural stability and joint position sense on the involved and uninvolved sides in patients with acute and chronic ACL tears. (a) Overall stability index (OSI). (b) Anterior-posterior stability index (APSI). (c) Mediolateral stability index (MLSI). (d) Reproduction of passive position (RPP). *p<0.05.

All three stability indices, OSI, APSI, and MLSI, on both the involved and uninvolved sides, were similar in the acute ACL and chronic ACL tear groups (Table 3). RPP of the involved side was significantly greater in the chronic than the in acute ACL tear group (7.84° ± 4.22° vs. 5.58° ± 4.31°, P = 0.041), although RPP of the uninvolved side was similar in these two groups (5.60° ± 4.95° vs. 6.74° ± 5.77°, P = 0.388, Table 3). In the acute ACL tear group, all stability indices, including OSI, APSI, and MLSI, and RPP, were similar on the involved and uninvolved sides, whereas, in the chronic ACL tear group, all stability indices except for MLSI were significantly greater on the involved than on the uninvolved side (Fig 1A–1D).

**Table 3:** Parameters of postural stability and joint position sense in patients with acute and chronic anterior cruciate ligament tears.

	Acute ACL group	Chronic ACL group	*P*-value
OSI uninvolved	1.45 ± 0.74	1.36 ± 0.59	0.620
OSI involved	1.65 ± 0.82	1.70 ± 0.63	0.778
APSI uninvolved	1.24 ± 0.59	1.25 ± 0.58	0.986
APSI involved	1.47 ± 0.84	1.51 ± 0.63	0.815
MLSI uninvolved	0.93 ± 0.37	0.79 ± 0.32	0.139
MLSI involved	0.89 ± 0.53	0.95 ± 0.39	0.605
RPP uninvolved	5.60 ± 4.95	6.74 ± 5.77	0.388
RPP involved	5.58 ± 4.31	7.84 ± 4.22	**0.041** *

Abbreviations: ACL, anterior cruciate ligament; OSI, overall stability index; APSI, anteroposterior stability index; MLSI, mediolateral stability index; RPP, reproduction of passive positioning

Results reported as mean ± SD.

*P< 0.05

### Correlations and Predictors of Reproduction of Passive Positioning of the Involved Limb

Of the parameters associated with proprioception and postural stability, only RPP on the involved side differed significantly between the acute and chronic ACL tear groups. Therefore, correlation analyses were performed between various parameters and RPP on the involved side. Univariate analysis showed that patient age, time from injury to surgery, peak torques of the hamstring muscles on the involved and uninvolved sides, and RPP on the uninvolved side were significantly correlated with RPP on the involved side (Table 4). Multiple linear regression analysis of these 5 parameters showed that duration from injury to operation (*β* = 0.202, *P* = 0.039) and RPP on the uninvolved side (*β* = 0.242, *P* = 0.026) were significant and independent predictors of RPP on the involved side (Table 5).

**Table 4:** Correlation between parameters and mean reproduction of passive positioning of the involved limb.

	RPP (involved)	
Parameters	Correlation coefficient	*p*-value
Age	0.348	**0.012** *
Height	-0.188	0.105
Weight	-0.106	0.364
Duration	0.332	**0.003** *
Quadriceps torque (uninvolved)	-0.177	0.127
Quadriceps torque (involved)	-0.159	0.169
Hamstring torque (uninvolved)	-0.251	**0.029** *
Hamstring torque (involved)	-0.247	**0.032** *
HQ ratio (uninvolved)	-0.175	0.129
HQ ratio (involved)	-0.164	0.158
OSI uninvolved	0.085	0.465
OSI involved	0.016	0.890
APSI uninvolved	0.119	0.304
APSI involved	0.012	0.916
MLSI uninvolved	0.023	0.847
MLSI involved	0.159	0.170
RPP uninvolved	0.310	**0.006** *

RPP, reproduction of passive positioning; H, hamstring; Q, quadriceps; OSI, overall stability index; APSI, anteroposterior stability index; MLSI, mediolateral stability index

*P< 0.05

**Table 5:** Multiple linear regression analysis of predictors of the reproduction of passive positioning of involved limbs in patients with anterior cruciate ligament tears.

Dependentvariable	IndependentVariables	Unstandardized coefficients		Standardized coefficients	
		B	SE (B)	B	P-value
RPP involved	Age	0.094	0.072	0.159	0.195
	Duration	0.079	0.044	0.202	**0.039** *
	RPP uninvolved	0.315	0.138	0.242	**0.026** *
	Hamstring torque (uninvolved)	-0.022	0.029	-0.091	0.460
	Hamstring torque (involved)	-0.031	0.029	-0.126	0.281

RPP, reproduction of passive positioning

*P< 0.05

## Discussion

This study quantitatively compared proprioception and postural stability in patients with acute and chronic ACL tears using parameters such as RPP and stability indices. The main finding of this study was that RPP on the involved side was greater in patients with chronic than acute ACL tears. In addition, RPP and the OSI and APSI stability indices were greater on the involved than on the uninvolved side, but only in the chronic ACL tear group.

Previous histologic and clinical studies[18–20] have yielded inconsistent results regarding whether a longer time from injury to surgery resulted in a deterioration of proprioception in ACL deficient knees. A study of arthroscopically obtained biopsy specimens of ACL remnant tissue from 20 patients with ACL tears found that morphologically normal mechanoreceptors remained in the ligament for three months after the injury, after which the number of mechanoreceptors gradually decreased. [19] By the ninth month after injury, only a few Pacinian corpuscles and free nerve endings were present, with these completely disappeared after 12 months. In contrast, assessments of proprioception, measured as threshold to detect passive motion (TTDPM), in 11 patients with complete ACL tears showed that the mean TTDPM difference was significantly higher in these patients than in an age-matched control group, indicating a greater decrease in proprioception in ACL deficient than in normal knees.[18] Although TTDPM of the involved and uninvolved sides did not differ between patients with acute and chronic tears, these results were not the primary outcome of the study, and the cutoff between acute and chronic ACL tears was not defined. We found that all stability indices on both the involved and uninvolved sides were similar in the acute and chronic ACL tear groups, with only RPP of the involved side being significantly greater in the chronic than in the acute ACL tear group. Furthermore, although we observed no differences in stability indices and RPP on the involved and uninvolved sides in the acute ACL tear group, all of these parameters, except for MLSI, were greater on the involved than on the uninvolved side in patients with chronic ACL tears. We also found that the time interval from initial injury was a predictor of RPP on the involved side. These findings suggested that the deterioration of proprioception of ACL deficient knees was dependent on time following injury. The disparity of results among studies may be due to a lack of standard tests of knee joint proprioception,[21] making it difficult to compare study results directly.

In addition to time following injury, we found that RPP on the uninvolved side was a significant predictor of RPP on the involved side, indicating a bilateral proprioceptive defect in patients with unilateral ACL tears.[22] Aberrant afferent information in the intra-articular receptors in the injured limb may also affect proprioceptionin the contralateral, uninjured limb. This bilateral impairment of proprioception may explain why the RPP of the uninvolved side significantly affected RPP on the involved side.

Previous studies have yielded conflicting results on the correlation between proprioception and postural stability. Because sensory information associated with a patient’s conscious perception of joint motion via mechanoreceptors in the ACL may contribute to postural stability, it can be inferred that a decrease of proprioception due to mechanoreceptor damage as a result of ACL tear may be related to a reduction of postural stability. A recent study of 10 chronic ACL deficient knees found a significant positive correlation between the TTDPM and dynamic stance stability on the involved side.[14] However, another study of 36 patients with chronic ACL tears found no correlation between TTDPM and postural stability in single limb stance.[23] Similarly, we observed no correlation between RPP and stability indices on the involved side, including OSI, APSI, and MLSI. There are several possible explanations for the inconsistent results reported for the correlation between proprioception and postural stability. Postural stability requires input of information, primarily from quick-adapting mechanoreceptors such as Pacinian corpuscles, whereas joint position sense is mediated primarily by slow-adapting mechanoreceptors such as Ruffini endings and Golgi tendon organs.[24, 25] The involvement of different mechanoreceptors in postural stability and joint position sense may explain the lack of correlation between RPP and stability indices. In addition, the different positions used for measuring proprioception (non weight bearing) and postural stability (weight bearing), that is, with and without the contribution of receptors in the feet and leg muscles, may give rise to different relationships between proprioception and postural stability. Finally, the influence of proprioceptive deficiency after ACL tear on postural stability may be offset by compensation from visual, vestibular, and somatosensory inputs in the joint structures around the knee.[26]

This study had several limitations. We were unable to measure the threshold to detect passive motion (TTDPM), a more sensitive method for quantifying proprioception than RPP. However, the experimental conditions and equipment used would have greater effect on TTDPM than on RPP.[27] Moreover, the test-retest reliability of RPP in our study was satisfactory. Another limitation was our inability to ensure that the differences in joint position sense between the acute and chronic ACL tear groups and between the involved and uninvolved sides were due entirely to damage to mechanoreceptors in the ruptured ACL remnant.[22] The capsular receptors and nerves may have been damaged at the time of initial injury, thus contributing to loss of proprioception.[28]

## Conclusions

The joint position sense on the affected side was worse in patients with chronic than with acute ACL tears, although there was no difference in postural stability between the two groups. In patients with chronic ACL tears, joint position sense and postural stability, except in the medio-lateral direction, were worse on the involved than on the uninvolved side. These differences, however, were not observed in patients with acute ACL tears. Taken together, these findings indicate that a longer elapsed time from injury to surgery results in worse proprioception and postural stability in ACL deficient knees. Therefore, to prevent loss of proprioception, ACL reconstruction should be performed as soon as possible after knee swelling has subsided and range of motion has been regained.

## Supporting Information

S1 Dataset Raw data used in this study. (XLSX)
